# Remediation: Streamside Solution

**DOI:** 10.1289/ehp.113-a156

**Published:** 2005-03

**Authors:** Lance Frazer

There are more than 3 million miles of streams in the United States, forming a significant part of our total watershed. Human activity frequently results in streamside vegetation being cut back or damaged, which can destabilize the stream bank and bury algal and microorganism populations under eroded and deposited sediment. It can also increase the amount of light falling on a stream, potentially increasing algal growth. A recent report suggests there are more benefits to keeping streamside vegetation intact than meet the eye. This greenery also appears to play a critical role in removing contaminants from stream waters.

“When you look at a stream,” says Oak Ridge National Laboratory scientist Patrick Mulholland, “your overriding impression centers on the flow. To the casual observer, it seems that whatever is in the stream, or gets dumped into it, just gets carried along to the next large body of water. It’s only recently we’ve begun to realize that isn’t accurate.”

In the September 2004 issue of *Biogeochemistry*, Mulholland reported on a 12-year study of the West Fork of Walker Branch, a small stream in eastern Tennessee. His study to date has revealed that there’s a tremendous amount of biological activity going on in streams, and that streamside vegetation plays a key role. According to Mulholland, a healthy growth of streamside vegetation can diminish the nitrogen and phosphorus load in the stream to the point that it reduces the risk of the nitrogen-driven algal blooms that plague many of the country’s lakes and coastal waters. He explains that the biological activity of small streams, because of their higher surface-to-volume ratio, takes place at a greater rate than that of larger streams.

The biological processes in a stream are driven by two factors: the sun, which provides energy for algae and bryophytes such as mosses, and the vegetation debris—the leaves, branches, and logs that fall into the stream—which provides energy for the microorganisms that remove the nitrogen and phosphorus from the stream water. Larger pieces of debris also help trap smaller debris particles, further enhancing the community.

Over the 12-year study, Mulholland says, these in-stream processes removed an average of 20% of the nitrate and 30% of the dissolved phosphorus entering the stream from the surrounding catchment. These common pollutants often enter the stream through inputs of sewage or lawn and farm fertilizer, and both may also be deposited atmospherically. Mulholland’s study indicated a much lower level of nitrate and dissolved phosphorus during the autumn and spring as a result of uptake by organisms within the stream. In fact, he says, November showed the greatest uptake of these pollutants, probably driven by microbes colonizing the newly fallen autumn leaves.

Streamside areas can and should be replanted, Mulholland says, but with some care. It is easier and cheaper to plant banks with short grasses rather than woody native vegetation, he says, but while grasses might help stabilize the bank to some degree—although less so than trees and shrubs will—they won’t add the needed organic material. “Best is to regrow the natural vegetation,” Mulholland says.

There is a very close relationship between healthy streamside vegetation and the health of the waterway, agrees Margaret Palmer, a professor of biology and entomology at the University of Maryland. “The problems we’re having with areas like the Everglades, the Great Lakes, and the Chesapeake Bay are all a function of what moves downstream. The microbes [Mulholland] is researching play a tremendous role—they remove nutrients from the water, they help break down toxics like petroleum byproducts, some even help fix heavy metals and remove them from the water. We have to preserve and restore these stream-side areas, not because it looks good, but because it will directly impact the quality of our lives.”

Palmer points out that the riverside vegetation itself also helps remove nutrients, as do the microbes in the soil of the stream banks. This complex system of nutrient processing—and not just in-stream processing—leads to the best water quality, she says.

We’re coming to realize that streamside vegetation is important in ways far beyond its appeal to our sense of aesthetics, Mulholland says. “We have a responsibility to maintain the ecological health of the stream system,” he asserts. “In many ways, the lands bordering our streams are among the most important part of our landscape.”

## Figures and Tables

**Figure:**
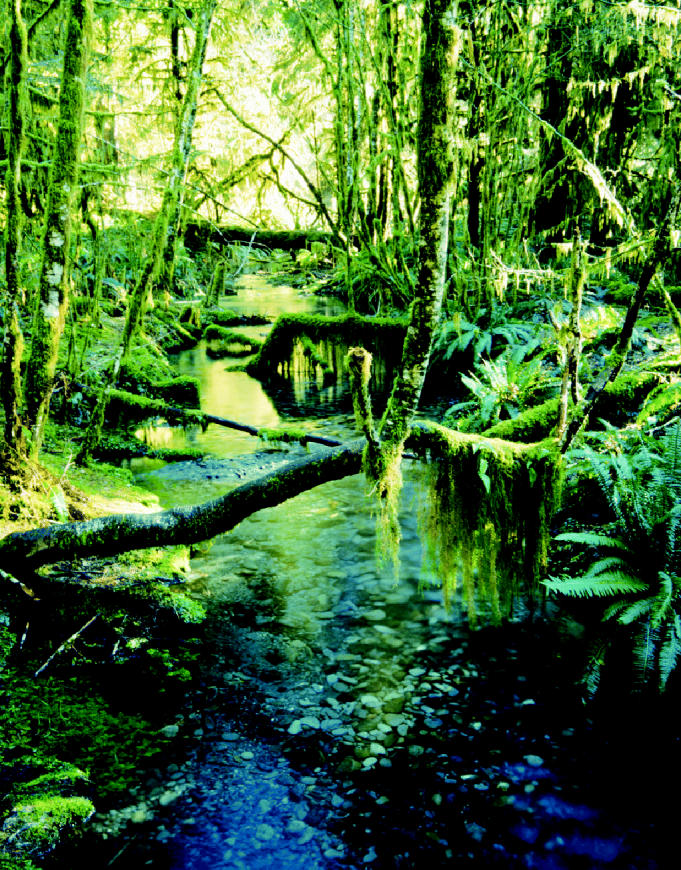
**Bank on it.** Results of a 12-year study show that renewing streamside vegetation may be the best bet for removing chemicals from streams.

